# Development of a Quality Score for the Home Food Environment Using the Home-IDEA2 and the Healthy Eating Index-2010

**DOI:** 10.3390/nu11020372

**Published:** 2019-02-12

**Authors:** Sarah K. Hibbs-Shipp, Richard E. Boles, Susan L. Johnson, Morgan L. McCloskey, Savannah Hobbs, Laura L. Bellows

**Affiliations:** 1 Department of Food Science and Human Nutrition, Colorado State University, Fort Collins, CO 80523, USA; sarah.hibbs-shipp@colostate.edu (S.K.H.-S.); morgan.mccloskey@colostate.edu (M.L.M.); savannah.hobbs@colostate.edu (S.H.); 2 Department of Pediatrics, University of Colorado Anschutz Medical Campus, Aurora, CO 80045, USA; Richard.boles@ucdenver.edu (R.E.B.); Susan.Johnson@ucdenver.edu (S.L.J.)

**Keywords:** home food environment, Healthy Eating Index, dietary quality, validation, psychometric

## Abstract

The home food environment (HFE) is an important factor in the development of food preferences and habits in young children, and the availability of foods within the home reflects dietary intake in both adults and children. Therefore, it is important to consider the holistic quality of the HFE. The purpose of this study was to apply the Healthy Eating Index (HEI; a measure of diet quality in conformance to the Dietary Guidelines for Americans) algorithm to the Home-IDEA2, a valid and reliable food inventory checklist, to develop a Home-IDEA2 HEI Score. After an initial score was developed, it was psychometrically tested for content, criterion, and construct validity. Content validity testing resulted in 104 foods being retained. Internal criterion testing demonstrated that 42 foods (40%) changed component scores by >5%; however, no single food changed a total Home-IDEA2 HEI score by >5%. Testing of hypothetical HFEs resulted in a range of scores in the expected directions, establishing sensitivity to varied HFEs. This study resulted in a validated methodology to assess the overall quality of the HFE, thus contributing a novel approach for examining home food environments. Future research can test interventions modifying the HFE quality to improve individual dietary intake.

## 1. Introduction

In recent years, dietary research has expanded to assess not only the foods eaten, but also the context in which the food is eaten (e.g., at home versus away from home) [1,2,3] and where the food was obtained (e.g., fast-food [4], sit down restaurant [5], convenience store [6,7], grocery store [8,9], school/cafeteria [10], or vending machine [9]) [11,12]. One of these areas of expanding research is the home food environment (HFE), which provides context for individual and family dietary intake [13]. The HFE has received increasing attention as an important factor in the development of food preferences and habits in children, as a contributor to obesogenic environments, and as a modifiable factor for nutritional interventions, especially those targeting childhood obesity [13,14,15].

The HFE is influenced by factors that include food purchasing and preparation decisions, food availability, and food accessibility [16,17]. The HFE impacts children’s diet not only through examples of eating habits, but also through the actual foods that are readily available and accessible in the home [18,19]. The availability of foods within the home has been shown to reflect intake in both adults and children, and as such, provides a potential dietary intervention point [3]. Intervention targets for improving children’s dietary behaviors should focus on the availability and accessibility of a spectrum of foods, including increasing healthful foods as well as reducing foods and beverages that are energy-dense and nutrient poor [20].

This increasing recognition of the environmental context of dietary intake has led to a large increase in the number of tools available for assessing a given environment with regard to availability of foods. Often HFE tools were designed to fit the researchers’ immediate questions and were brief, focused on only one aspect of food availability—such as high-fat foods, sugar-sweetened beverages, or fruits & vegetables—and had limited psychometric testing performed [21]. Small food sets and a lack of a complete listing of foods in most HFE assessments has limited researchers’ abilities to examine the totality of the HFE and its contributions to dietary quality.

The Home Inventory to Describe Eating and Activity (Home-IDEA2) is one such assessment that has been found to be valid and reliable in assessing food, activity, and electronic home environments among low-income minority parents of preschoolers [22]. The Home-IDEA2 is a semi-comprehensive checklist designed to assess the foods present in the home at a single point in time. It includes foods sourced from the Allowable Foods List from the US Special Supplemental Nutrition Program for Women, Infants, and Children (WIC Program), the Block Food Frequency Questionnaire, and the modified Harvard Food Frequency Questionnaire (FFQ), allowing for application to diverse households.

Given the relationship between the HFE and dietary intake, it is important to consider the HFE beyond the presence and absence of individual foods, and examine the holistic quality of the entire HFE. The Healthy Eating Index (HEI), developed by the National Cancer Institute, is the method of choice in the US for assessing dietary quality [23]. The HEI is a formalized approach that includes rules and analysis algorithms allowing for effective comparisons in the overall quality of foods across different levels of the food supply [23,24]. HEI algorithms have been applied to the US food supply level [25], the community food environment (e.g., food assistance program offerings [26], supermarket sales circulars [27], menu offerings [4], corner stores [28], grocery purchases [29], by multiple food purchase locations [30]), and at the individual food intake level (e.g., comparing diet cost to diet quality [31], comparing different dietary patterns [32,33,34,35], and evaluating differences in mortality outcomes by diet quality [36]). To date, the HEI algorithm has been applied to the HFE in one recent study [37], which demonstrates a need for more rigorous validation and psychometric testing. Application of the HEI to the HFE provides a unique way of assessing overall quality of the HFE, and allows for direct comparison to dietary intake quality, thus providing the potential for further insights into the relationship between the HFE, dietary intake, and health outcomes.

For this study, the Home-IDEA2 was used as an instrument to apply the HEI algorithm to assess the quality of the HFE. Specific objectives were to (1) develop an initial Home-IDEA2 HEI Score, and (2) psychometrically test the Home-IDEA2 HEI Score for content, criterion, and construct validity.

## 2. Materials and Methods

### 2.1. Development of an Initial Home-IDEA2 Healthy Eating Index Score

#### 2.1.1. Application of the HEI Algorithm to the Home-IDEA2

The Healthy Eating Index (HEI) is updated to conform to each edition of the Dietary Guidelines for Americans (DGA), with the HEI-2010 [23] reflecting diet patterning in conformance with the 2010-DGAs [38]. The HEI-2010 was used, as the HEI-2015 was not yet available during the development of the Home-IDEA2 HEI Score. Briefly, the HEI-2010 scores 12 dietary components for a total score ranging from 0–100 (Table 1), with total scores less than 51 categorized as ‘poor,’ 51–80 as ‘needs improvement,’ and greater than 80 as ‘good’ [39]. The HEI has been applied to other food supply levels using three steps: (1) identification of a set of foods, (2) determination of the amount of each dietary constituent associated with each food in the set, and (3) deriving ratios to score each HEI component using developed algorithms [40].

The Home-IDEA2 (the self-report checklist that participants complete regarding availability of select food items in the home) includes 108 food items that represent a wide variety of potential types of foods in the home. For example, there are single-items, such as “apple,” that represent all types of raw apples (Granny Smith, Macintosh, Red Delicious). There are also composite items that represent a “category” of similar items, such as “citrus fruits” representing oranges, tangerines, mandarins, grapefruit, lemons, limes, etc. All items, whether single or composite, are completed in terms of “Yes/No” availability in the home. No information is obtained as to how much of these items are in the home, rather, the presence or absence of the listed foods is assessed.

**Figure 1:**
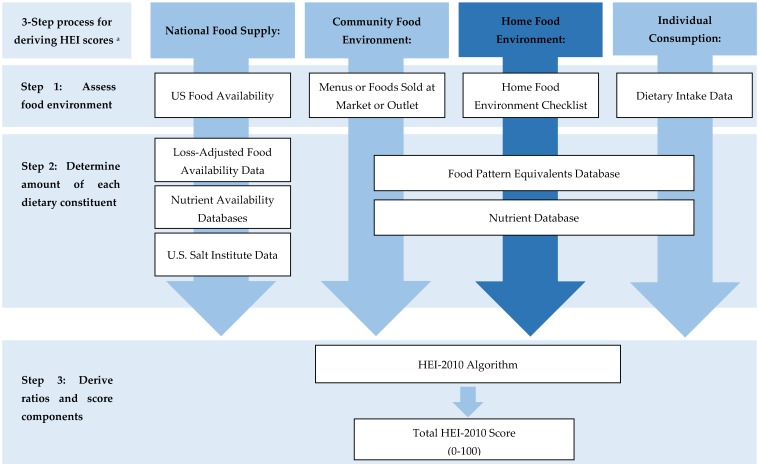
Three step process for deriving Healthy Eating Index (HEI) scores across different levels of the food system. ^a^ Figure adapted from Calculating HEI Scores at Different Levels, the HEI scoring illustration [45].

The Home-IDEA2 was chosen as the basis for developing a HFE quality score using the HEI for four reasons: (1) the high feasibility for individuals to complete the survey [22], (2) the included foods are relevant to socioeconomically, racially/ethnically, and geographically diverse families with young children [43,44], (3) it has been psychometrically validated [22,44], and (4) it has demonstrated positive associations with dietary intake for a broad range of foods ranging from healthy and less healthy foods in families with young children [43]. In order to apply the HEI to the Home-IDEA2, USDA’s Center for Nutrition Policy and Promotion’s recommended processes were modified to include the HFE, and a 3 step process was followed (Figure 1) [40]. Because all foods in the Home-IDEA2 are listed generically and without amounts, a ‘representative’ food identifying a specific food code that links to the Food and Nutrition Database for Dietary Studies (FNDDS) and a representative food amount for each Home-IDEA2 item needed to be assigned to apply nutrient values before ratios and HEI score components could be derived.

#### 2.1.2. Representative Foods

The representative foods and food amounts for the Home-IDEA2 items were sourced from the FoodAPS, a national survey of 4826 ethnically and income-diverse U.S. households conducted by the USDA Economic Research Service (ERS) and Food and Nutrition Service (FNS) between April 2012 and January 2013 [46]. The publicly available, de-identified, food-at-home dataset was used for this study (faps_fahnutrients, downloaded 26 January 2017) [46]. A multi-part process was employed to identify a representative food for each Home-IDEA2 item. First, a keyword search within the FoodAPS file was conducted for foods that matched each Home-IDEA2 item. Next, four investigators with expertise in nutrition and psychometric testing evaluated the identified foods for face validity with Home-IDEA2 items and reasonableness for low-income, multi-ethnic households. These foods were discussed by the research team until consensus was achieved. Last, remaining options were evaluated for key nutrients/nutrient categories (e.g., sodium, whole fruit, whole grains) theorized to load into the HEI-2010 algorithm, with the food closest to the mean or median of the key nutrients/nutrient categories selected as the ‘representative’ food for each Home-IDEA2 item. Table 2 outlines key considerations for the selection of the representative food for citrus fruits.

#### 2.1.3. Food Amounts

For each representative food, food amounts were then selected. Within the FoodAPS dataset, the mean, median, and mode of available total edible gram weights were calculated for each representative food. Next, an internet search for standard consumer package sizes was performed. Calculated weights were adjusted to reflect reasonable package sizes for consistency across foods (e.g., milk varieties (whole, 2%, 1% skim, chocolate) were normalized to 1 gallon (3.8 L), cheese varieties (regular, low-fat) were normalized to 1 pound (454 g), meat varieties (chicken, beef) were normalized to 2 pounds (908 g), and for realistic purchase quantities (e.g., vegetable oil was reduced from 1 gallon (3.8 L) to 32 ounces (1 L)).

#### 2.1.4. Ratios and HEI Score Components

The nutritional content for the representative foods and selected food amounts were merged with the Home-IDEA2 to create the Home-IDEA2 HEI Score database. The Home-IDEA2 captures a snapshot of the home at a single point in time, similar to a single dietary recall for one person; therefore, the HEI algorithm selected was “Calculating an individual’s HEI-2010 score, using FPED, and one day of 24HR recall” [47]. Two nutrient files were created, mirroring the layout of individual dietary intake nutrient analysis files obtained from the Automated Self-Administered 24-h Recall System (ASA24), the INFMYPHEI (Items/Individuals Foods and Pyramid Equivalents Data), and TNMYPHEI (Total/Daily Total Nutrient and Pyramid Equivalents Data) files [48]. The algorithm was then applied to the Home-IDEA2 HEI Score database to generate HEI component and total scores.

### 2.2. Psychometric Testing on the Home-IDEA2 HEI Score

Extensive validation and psychometric testing was performed to test the functionality of the Home-IDEA2 HEI Score. Face validity was assessed throughout the selection of the representative foods and food amounts. Decisions were made to match the intention of Home-IDEA2 items and to control for foods that might reasonably be found in participants’ homes. Content, internal criterion, and construct validity were also tested (Table 3).

#### 2.2.1. Content and Criterion Validity

Content and internal criterion validity were examined through over 300 rounds of iterative testing in the application of the HEI algorithm to the Home-IDEA2 HEI Score database. Iterative testing involved the removal of each food from the Home-IDEA2 HEI Score database to determine if the representative food was loading into the component scores as theorized (content validity), and to test the individual and cumulative group contributions of each food to component and total scores (internal criterion validity). The effect size was calculated by the percent change in both the component and total score after the removal of each individual food item from the total pattern. Due to the patterning nature of the HEI algorithm, it was necessary to test for inappropriately high effects of individual representative food loadings on component and total scores.

#### 2.2.2. Construct Validity

Construct validity was assessed with five sample Home-IDEA2 checklists that were created to represent various diet patterns ranging from minimally healthful (theorized low HEI score) to very healthful (theorized high HEI score). These patterns included a highly processed pattern, a moderately processed pattern, a vegetarian pattern with minimal processed foods, and two dietary patterns that were used as evidence-based referent groups: the DASH diet [49], and a pattern based on the Dietary Guidelines for Americans (DGA) [50]. To test adherence to the DGA, a Child and Adult Care Food Program (CACFP) weekly menu was used to create the sample checklist. These food patterns were selected to examine sensitivity, and to evaluate if our tool and the resulting quality score would produce different scores for different home food environments. All analyses were conducted using SAS (version 9.4; SAS Institute Inc., Cary, NC, USA). The HEI-2010 algorithm was provided by the National Cancer Institute [40].

## 3. Results

### 3.1. Development of an Initial Home-IDEA2 HEI Score

In the process of determining representative foods from the FoodAPS, two Home-IDEA2 items were eliminated. “Unprepared mixes” was eliminated due to the complexity of options available, which did not allow for an accurate selection of a single representative food, and there were no options for “tortilla, other” outside of corn or flour, which were already captured as individual food items. In the process of determining food amounts, two additional Home-IDEA2 items were removed due to a lack of total edible gram (TEG) weights (rice cakes), and a TEG weight that had no comparable consumer purchase size (deer—the TEG from the FoodAPS database represented an entire deer carcass), leaving 104 foods in the Home-IDEA2 HEI Score database.

### 3.2. Psychometric Testing on the Home-IDEA2 HEI Score

#### 3.2.1. Content and Criterion Validity

In testing content validity, results from iterative testing showed that two foods (those representing chocolate/candy and unsweetened cereal) did not load into component scores as initially hypothesized. Therefore, changes were made to the representative foods initially selected for chocolate/candy and unsweetened cereal to correct component score loading and maintain the original intent of the food within the Home-IDEA2. Inconsistent effects in component outcomes were also observed for processed food items and cooking oils/fats. Food amounts were adjusted to create similar effect sizes on component scores within each food category (e.g., fruits, processed foods, grains, cooking oils).

Iterative testing was then repeated to confirm changes in effect sizes for component and total scores. Internal criterion validity was demonstrated, as each representative food had larger percentage effect sizes in the relevant component score(s) than in the total score (Table 4). Five foods—ramen, brown rice, broccoli, grapes, and vegetable oil—were highlighted, as they were the foods in the Home-IDEA2 HEI Score database that had the largest impact in a given component score: e.g., Sodium, Whole Grain, Whole Fruit, and Fatty Acid Ratios, respectively. Despite larger changes to component scores, there was no single food that resulted in a change of greater than 5% to the total score. For example, the absence of broccoli from a household yielded a 21.1% negative change to the Greens and Beans component score, but only a negative 1.2% change to the total score. Of the 104 foods in the Home-IDEA2 HEI Score database, 42 affected a change of at least 5% in one or more component scores when removed from analysis. Of those 42 foods, 13 affected a 10–20% change, with 2 affecting over a 20% change (broccoli: −21.1% change in Greens and Beans; vegetable oil: −31.1% change in Fatty Acid Ratio). This demonstrated internal criterion validity with regard to the intent of the algorithm (i.e., component scores represent individual food contribution, whereas the total score represents the overall patterning) [24].

#### 3.2.2. Construct Validity

The analyses of the five sample HFEs resulted in a range of scores in the expected directions for both component and total scores (Table 5). The highest total scores (out of 100) resulted from the minimally processed/vegetarian (93.8) and DASH (88.9) HFEs and are classified as ‘good’ (total score >80) according to the Center for Nutrition Policy and Promotion’s standardized guidelines for HEI scores [39]. The CACFP HFE resulted in a nearly ‘good’ score, 78.9, with low scores for the seafood and plant proteins and fatty acid ratio components, as CACFP menus used to create the sample HFE did not include any food items that would contribute to the seafood and plant protein component. Moreover, while the CACFP menus contained substantial dairy (contributing saturated fatty acids to the denominator), they did not include foods that contained MUFAs and PUFAs (the numerator) for the fatty acid ratio component. All other component scores, excluding sodium, were maximized by the CACFP environment, thus indicating a high ability to detect adherence to the DGAs within the bounds of using the Home-IDEA2. The moderately and highly processed HFEs scored lower for most component scores and generated lower total scores (in the ‘needs improvement’ category and close to ‘poor’) than the more healthful HFEs, suggesting measurement sensitivity to different patterns in the anticipated directions.

To further examine construct validity, broccoli was included in all five sample HFE patterns; broccoli is the only vegetable in the Home-IDEA2 HEI Score database that contributed to the Greens and Beans component. The Highly Processed sample HFE had non-maximum scores for the Greens and Beans component, whereas the Minimally Processed household, DASH household, and CACFP households scored the maximum of 5. This demonstrated that the presence of a single food within the total patterning of a given household may result in a range of scores within a component.

## 4. Discussion

In this study, a HFE quality score was developed by applying the HEI to the Home-IDEA2, a validated assessment of the HFE. The development process used to produce the Home-IDEA2 HEI Score mirrored those employed for the HEI-2010 [24] and, therefore, utilized a validated method in which an index of dietary quality was quantitatively applied to foods available in the home environment. Because the HFE is a significant target for efforts focused on prevention of childhood obesity, enhancing researchers’ ability to measure multiple environmental correlates, including availability, accessibility, and quality of foods, is critical to understanding children’s food behaviors and dietary intake.

Having a comprehensive measure of HFE overall quality in addition to dietary intake quality provides a more complete picture of how the HFE may impact dietary intake at the pattern level, thus aligning HFE research with current trends in dietary intake research examining dietary patterning in addition to individual food groups or nutrients [51,52,53,54]. Further, having a validated Home-IDEA2 HEI Score provides several important opportunities for researchers. The Home-IDEA2 HEI Score can be used not only to easily summarize food quality in the home, but to measure the overall quality of the home food environment as an intervention target. Similarly, the score could be used in a larger cohort study for assessing the associations between the quality of the HFE and health outcomes.

Extensive steps were taken to validate the Home-IDEA2 HEI Score at each step of the development process. Examining the relative percent change of individual foods to component and total scores confirmed that the vast majority of representative foods had negligible impact on the total score when considered individually. Because the HEI is designed to measure overall patterning, this was critical, as it demonstrated that no individual food had the power to significantly impact the total score. It should be noted that changes in the presence or absence of foods may reflect changes in the overall categorization of a home’s Home-IDEA2 HEI Score as poor (less than 51), needs improvement (51–80), or good (greater than 80) [39]. For example, as Ramen induced a 3.4% change to the total score, it is feasible that a home’s overall Home-IDEA2 HEI Score might move above or below these prescribed categories. Construct validity was demonstrated through testing of five sample HFEs, which revealed both total and component scores in the expected directions. Similarly, this analysis resulted in a range of total scores for each of the five sample HFEs (56.0–93.8), indicating that scores ranged from nearly all three standardized HEI rating quality categories: poor, needs improvement, and good [39].

The process of applying the HEI algorithm to the Home-IDEA2 revealed opportunities for improvement of the checklist. First, the Home-IDEA2 was not designed with the HEI in mind, thus, the retrospective application of the HEI identified gaps in the food items included in the checklist. The Home-IDEA2 was unbalanced, with a greater variety and higher number of more healthful foods feeding into the HEI components that make up the adequacy score, compared with fewer options for less healthful/processed food items that contribute to the HEI components contributing to the moderation score. This is highlighted by the Home-IDEA HEI score (Table 5) of 75.2 (out of 100) when all foods were checked as present in the home. Ideally, when both healthful (adequacy) and unhealthful (moderation) foods are adequately represented, the total HEI score should be lower. Future iterations of the checklist should include more options of moderation foods. Similarly, future versions of the Home-IDEA should be designed with the HEI in mind and include multiple food types that represent HEI components (so that the presence of one food, like broccoli, does not create a perfect component score). Further understanding of how the presence or absence of these foods in the home might affect the Home-IDEA2 HEI Score, and whether or not equal representation is given to all food components of the HEI, is warranted.

While the HEI is designed to measure adherence to the U.S. Dietary Guidelines, algorithms for other dietary patterns, such as the DASH diet, the Mediterranean diet, the Alternative HEI, and country/region specific dietary guidelines, could be applied in a similar manner as the HEI to the Home-IDEA2. The process of applying a dietary pattern algorithm to the HFE to develop a quality score has the potential to be replicated to encompass culturally specific eating patterns, or could be used to compare and contrast HFE across countries/regions. Culturally tailoring the Home-IDEA2 or the application of the dietary pattern algorithms to different HFE assessment tools would require content, criterion, and construct validity testing to ensure that the tool and the algorithm were reflecting the intent of the dietary pattern appropriately.

There were limitations and strengths to this study. As mentioned, the predetermined list of foods reduced participant and researcher burden, but the Home-IDEA2 is not all-inclusive and may place limits on capturing the full diversity of foods in the home. The Home-IDEA2 is similar to a single dietary recall for one person, and as such, is subject to the same limitations, such as substantial day-to-day variability. While the checklist has been validated and successfully used in low-income, multi-ethnic families with young children [43,44], food items cover a large portion of foods frequently consumed by all Americans, and future research with other audiences is warranted. The food categories of the Home-IDEA2 were assigned representative foods for nutrient content and food amount using the FoodAPS database, a nationally representative database of U.S. households. However, it should be noted that the selection of representative foods (face validity) was completed by the experts in nutrition and psychometric testing, and, therefore, the subjectivity of these selections is a limitation. Finally, the development and validation procedures for the Home-IDEA2 HEI Score were modeled after USDA recommendations. Extensive validation procedures and psychometric testing were used to ensure that the Home-IDEA2 HEI Score was functioning as intended.

## 5. Conclusions

The ability to assess the quality of the foods in the home holistically via the application of an HEI score allows for a more complete view of the HFE, and provides a useful form of measurement to future observational and intervention studies working to gain a fuller understanding of the HFE. Previous literature has shown the importance of understanding and measuring the HFE, because of the predictive ability of the availability of food in the home to the types of foods that children eat [16,17,18]. This study adds to the literature a psychometrically tested and thoroughly validated measure. While this study used an existing instrument, future modifications to the Home-IDEA2 are needed to address the aforementioned limitations, to improve sensitivity and enhance the ability to accurately measure the overall quality of the HFE with a constrained number of food items. The ability to quantify HFE quality in a valid way provides researchers with a methodology to holistically assess families and children’s food environments, which may contribute to a greater understanding of dietary intake and, ultimately, health outcomes.

## Figures and Tables

**Table 1:** Healthy Eating Index-2010 components and scoring standards.

HEI-2010 Components	Maximum Points	Standard for Maximum Score (per 1000 kcal) ^a^	Standard for Minimum Score of Zero (per 1000 kcal) ^a^
Adequacy			
Total Fruit ^b^	5	≥0.8 cup equiv. (102 g)	No Fruit
Whole Fruit ^c^	5	≥0.4 cup equiv. (51 g)	No Whole Fruit
Total Vegetables ^d^	5	≥1.1 cup equiv. (141 g)	No Vegetables
Greens & Beans ^d^	5	≥0.2 cup equiv. (26 g)	No Dark Green Vegetables or Beans/Peas
Whole Grains	10	≥1.5 oz equiv. (42 g)	No Whole Grains
Dairy ^e^	10	≥1.3 cup equiv. (166 g)	No Dairy
Total Protein Foods ^f^	5	≥2.5 oz equiv. (71 g)	No Protein Foods
Seafood & Plant Proteins ^f,g^	5	≥0.8 oz equiv. (23 g)	No Seafood or Plant Proteins
Fatty Acid Ratio ^h^	10	(PUFAs + MUFAs)/SFA ≥2.5	(PUFAs + MUFAs)/SFA ≤1.2
Moderation			
Refined Grains	10	≤1.8 oz equiv. (~51.0 g)	≥4.3 oz equiv. (122 g)
Sodium	10	≤1.1 g	≥2.0 g
Empty Calories ^i^	20	≤19% of energy	≥50% of energy
Total Score ^j^	100		

Adapted from the National Cancer Institute’s HEI-2010 Components & Scoring Standards Table [41]. (Development of the scoring rubric has been previously described in detail [42]. **^a^** Amounts between the minimum and maximum scores are scored proportionately. ^b^ Includes 100% fruit juice. ^c^ Includes all forms except juice. ^d^ Includes any beans and peas not counted as total protein foods. ^e^ Includes all milk products, such as fluid milk, yogurt, and cheese, and fortified soy beverages. ^f^ Beans and peas are included here (and not with vegetables) when the total protein foods standard is otherwise not met. ^g^ Includes seafood, nuts, seeds, and soy products (other than beverages), as well as beans and peas counted as total protein foods. ^h^ Ratio of poly- and monounsaturated fatty acids (PUFAS and MUFAS) to saturated fatty acids (SFAs): (PUFAs + MUFAs)/SFA. ^i^ Calories from solid fats, alcohol, and added sugars; threshold for counting alcohol is >13 g/1000 kcal. ^j^ The Center for Nutrition Policy and Promotion categorizes total scores as poor (less than 51), needs improvement (51–80), and good (greater than 80) [39]. Abbreviations: HEI: Healthy Eating Index; equiv., equivalents; oz., ounces; g., grams.

**Table 2:** Sample considerations for the selection of the representative food for Home-IDEA2 Category of citrus fruits.

Potential Representative Food Item	HEI Component Score	Presence in Households ^a^	Availability, Consumption and Other Considerations	Selection Decision
Oranges	Whole fruit; Total fruit	Common	*Availability:* Year-round; *Consumed:* As whole fruit	Selected (food code 61119010)
Clementines (Cuties^®^)	Whole fruit; Total fruit	Common	*Availability:* Seasonal; *Consumed:* As whole fruit	Not selected due to seasonality
Mandarins	Whole fruit; Total fruit; SOFAAS (added sugars)	Common	*Availability:* Seasonal (fresh); Year Round (packaged); *Consumed:* As whole fruit; *Other:* Packed in juice and syrup; contains added sugars	Not selected due to contribution of added sugars
Grapefruit	Whole fruit; Total fruit; SOFAAS (added sugars)	Less common	*Availability:* Seasonal (fresh); Year Round (packaged); *Consumed:* As whole fruit; *Other:* Packed in juice and syrup; contains added sugars	Not selected due low presence in households and contribution of added sugars
Tangerine	Whole fruit; Total fruit	Less common	*Availability:* Seasonal; *Consumed:* As whole fruit	Not selected due to low presence in households and seasonality
Lemons	Whole fruit; Total fruit	Common	*Availability:* Year-round; *Consumed:* Not typically eaten in whole as fruit	Not selected because not typically consumed as whole fruit
Limes	Whole fruit; Total fruit	Common	*Availability:* Year-round; *Consumed:* Not typically eaten in whole as fruit	Not selected because not typically consumed as whole fruit

^a^ Presence in households was determined based on frequencies from Home-IDEA2 administered with a low-income, minority population (unpublished data). Abbreviations: HEI: Healthy Eating Index; Home-IDEA: Home Inventory Describing Eating and Activity; SoFAAS: Solid Fats, Alcohol, Added Sugars.

**Table 3:** Development and initial validation of Home-IDEA2 HEI Score.

	Validity Measure	Research Question	Analysis Strategy
Objective 1: Develop an initial Home-IDEA2 HEI Score	Face	Do the representative food items and amounts selected represent the intent of each Home-IDEA2 item? Would the representative foods be reasonably found in the target population homes?	Expert review of representative foods and food amounts, including comparison to standard consumer packaging sizes
Objective 2: Psychometric Testing of Home-IDEA2 HEI Score	Content	Do the representative foods feed into the HEI component and total scores as theorized?	Iterative runs of the HEI-2010 algorithm on the Home-IDEA2 HEI Score Database; each food was removed individually and changes in scores were visually examined
	Criterion	Are there any individual representative foods that impact HEI component or total scores more substantially than other foods?	Iterative runs of the HEI-2010 algorithm on the Home-IDEA2 HEI Score Database; each food was removed individually and changes in scores were visually examined
	Construct	Does the Home-IDEA2 HEI Score identify different home food environments?	Test the Home-IDEA2 HEI Score on five sample Home-IDEA2 checklists representing varying diet patterns (CACFP, DASH, vegetarian, moderately processed, highly processed)

**Table 4:** Criterion validity testing: percent (%) change values for HEI-2010 components and total score when specified food was removed from the Home-IDEA2 HEI Score database for 5 example foods.

	Percent (%) Change ^a^				
HEI-2010 Component	Ramen	Brown Rice	Broccoli	Grapes	Vegetable Oil
Adequacy					
Total Fruit	2.0	0.7	0.1	−5.3	5.5
Whole Fruit	3.3	1.2	0.1	−10.7	5.1
Total Vegetables	2.0	0.7	−3.8	0.3	5.4
Greens and Beans	0.7	0.3	−21.1	0.1	2.0
Whole Grains	2.2	−7.3	0.1	0.3	5.9
Dairy	2.6	0.9	0.1	0.3	7.0
Total Protein Foods	3.0	1.1	0.1	0.4	8.2
Seafood and Plant Proteins	0.0	0.0	0.0	0.0	0.0
Fatty Acid Ratio	4.2	-0.1	0.0	0.0	−31.1
Moderation					
Refined Grains	5.4	−1.4	−0.1	−0.5	−10.5
Sodium	11.4	−1.7	0.0	−0.6	−12.7
SoFAAS Calories	1.5	−0.9	-0.1	−0.3	−7.0
Total Score	3.4	−0.9	−1.2	−0.9	−4.2

^a^ Percent change was calculated relative to the maximum score for each component category, so the values presented are normalized to accurately reflect the correct weighting across categories. For example, if there was a change of 0.05 in a component with a maximum score of 5, the relative percent change is 1.0%, whereas a maximum score of 10 yields a percent change of 0.5%. Positive percent change values indicate that the component or total score has increased (become more aligned with the 2010 DGAs). Negative percent change values indicate that the component or total score has decreased (become less aligned with the 2010 DGAs). Abbreviations: HEI: Healthy Eating Index; Home-IDEA: Home Inventory Describing Eating and Activity; DGA: Dietary Guidelines for Americans; SoFAAS: Solid Fats, Alcohol, Added Sugars.

**Table 5:** Construct validity: five sample home food environments and resulting HEI-2010 component and total scores.

			Sample Home Food Environments				
HEI-2010 Components	Maximum Points	Home-IDEA2 (All Foods Present)	Highly Processed	Moderately Processed	Minimally Processed, Vegetarian	DASH	CACFP
Adequacy							
Total Fruit	5	2.9	2.0	3.0	4.4	3.3	5.0
Whole Fruit	5	4.7	3.7	4.1	5.0	3.6	5.0
Total Vegetables	5	2.8	2.0	3.4	5.0	5.0	5.0
Greens & Beans	5	1.1	2.1	0.0	5.0	5.0	5.0
Whole Grains	10	6.2	0.5	0.5	10.0	8.0	10.0
Dairy	10	7.4	6.1	4.4	4.4	4.0	9.1
Total Protein Foods	5	4.3	4.7	4.3	5.0	5.0	5.0
Seafood & Plant Proteins	5	5.0	5.0	2.6	5.0	5.0	0.0
Fatty Acid Ratio	10	8.1	6.5	10.0	10.0	10.0	0.0
Moderation							
Refined Grains	10	6.2	0.8	5.0	10.0	10.0	10.0
Sodium	10	8.9	6.9	7.7	10.0	10.0	6.4
Empty Calories	20	17.6	15.7	20.0	20.0	20.0	18.4
Total Score ^a^	100	75.2	56.0	64.9	93.8	88.9	78.9

^a^ The Center for Nutrition Policy and Promotion categorizes total scores as poor (less than 51), needs improvement (51–80), and good (greater than 80) [39]. Abbreviations: HEI: Healthy Eating Index; DASH, Dietary Approaches to Stop Hypertension; CACFP, Child and Adult Care Food Program.
